# Acclimatization response to a short-term heat wave during summer in lactating Brown Swiss and Holstein Friesian cows

**DOI:** 10.3389/fvets.2025.1582884

**Published:** 2025-04-28

**Authors:** Aristide Maggiolino, Lucrezia Forte, Alessandra Aloia, Umberto Bernabucci, Erminio Trevisi, Cristina Lecchi, Fabrizio Ceciliani, Geoffrey E. Dahl, Pasquale De Palo

**Affiliations:** ^1^Department of Veterinary Medicine, University of Bari A. Moro, Valenzano, Italy; ^2^Department of Agriculture and Forest Sciences (DAFNE), University of Tuscia, Viterbo, Italy; ^3^Department of Animal Sciences, Food and Nutrition (DIANA), Facoltà di Scienze Agrarie, Alimentari e Ambientali, Università Cattolica del Sacro Cuore, Piacenza, Italy; ^4^Department of Veterinary Medicine and Animal Science, Università degli Studi di Milano, Lodi, Italy; ^5^Department of Animal Sciences, University of Florida, Gainesville, FL, United States

**Keywords:** heat stress, Brown Swiss, Holstein Friesian, physiological patterns, milk

## Abstract

**Introduction:**

Dairy cows are highly susceptible to heat stress, raising concerns about animal welfare, production efficiency, and economic losses. Previous studies suggest that Holstein and Brown Swiss breeds exhibit different levels of thermal tolerance, but their short-term adaptive responses require further investigation.

**Methods:**

This study aimed to evaluate breed-specific physiological and productive responses to a 4-day natural heat wave in 40 lactating cows (20 Holstein, 20 Brown Swiss) from the same commercial dairy farm, homogeneous for days in milk, body condition score, parity, and energy-corrected milk yield. Before the heat wave, cows experienced at least 48 h in thermoneutral conditions. Physiological parameters were recorded three times daily (4:00 AM, 3:00 PM, and 8:00 PM). Blood samples were collected before the heat wave (D1, 4:00 AM, thermoneutral conditions) and at the warmest moment of the fourth day (D4, 3:00 PM, heat stress conditions).

**Results and Discussion:**

The heat wave negatively impacted physiological parameters in both breeds. Rectal temperature increased daily from 4:00 AM to 3:00 PM (*p* < 0.01), with Holstein cows showing consistently higher values than Brown Swiss (*p* < 0.01). Respiration rate reached its lowest point at 4:00 AM each day (*p* < 0.01) but remained elevated at 8:00 PM, despite decreasing THI, indicating accumulated heat load. While both breeds followed a similar trend, Holsteins exhibited a greater capacity for overnight recovery compared to Brown Swiss. Regarding productivity, Brown Swiss cows maintained stable milk yield (MY) from D1 to D4, whereas Holsteins showed a progressive MY decline throughout the heat wave (*p* < 0.01). Most blood parameters showed no significant breed differences (*p* > 0.05), but heat shock protein 70, a key regulator of thermal adaptation, exhibited an increasing trend in both breeds (*p* < 0.01), appearing earlier than other physiological indicators of heat stress.

**Conclusion:**

This study, conducted under identical conditions, highlights distinct breed-specific responses to short-term heat stress. The findings suggest that Brown Swiss cows may be more resilient to heat stress in terms of productivity, while Holsteins show better nighttime recovery. Further research should explore additional physiological and molecular markers to better characterize breed differences and improve heat stress mitigation strategies in dairy farming.

## 1 Introduction

Climate change poses a global challenge to the livestock industry, causing significant economic losses (1). Heat stress compromises livestock well-being, negatively impacting milk yield and quality (2). Heat stress occurs when animals fail to efficiently dissipate excess heat, leading to a rise in body temperature beyond the normal range, driving the animals to activate several mechanisms to maintain thermal balance (3). Thermal stress is influenced by various environmental factors, including dry bulb temperature, relative humidity, air movement, precipitation, atmospheric pressure, ultraviolet light, dust, and solar radiation, among others (4). As some of these factors may not be easily detectable, the most widely used indicator for thermal stress condition is the temperature humidity index (THI) (5). This bioclimatic index combines dry bulb temperature and relative humidity. Lactating cows are particularly affected by heat stress, given the metabolic heat load generated during milk production, coupled with the demands of nutrient digestion (2, 6). Performance is impacted by climatic stressors and the animal’s physiological adaptive mechanisms, which can enhance the overall losses (7). Heat tolerance is a complex phenomenon influenced by genetics (species, breed and individual variability), physiological status, management or production systems, and nutritional status, all of which can affect the animal’s response to heat stress (7–9). Deepening our understanding of the acclimatization responses of dairy cows is crucial for identifying biological markers that can quantitatively measure heat stress responses. These identified markers may then serve as valuable tools in breeding programs to select more thermo-tolerant breeds (10).

One of the well-known genetic factors affecting heat tolerance is the breed, in fact it is reported a higher resistance in Brown Swiss than Holstein cows (11, 12). Studies have been conducted on Italian Brown Swiss cows, where the identified threshold for the THI affecting productive patterns is 74 (2). In contrast, for Italian Holstein Friesian cows, the threshold at which the animals experience heat stress is slightly lower, at 72 (13). Several studies compared Holstein, Brown Swiss, and their crosses under subtropical environmental conditions. Abdalla and El-Tarabany (14) observed that reproductive performance was less affected by subtropical environmental conditions in Brown Swiss and their crosses compared with the pure Holstein Friesian breed. Other studies on sires (15) and lactating cows (11) emphasized the greater susceptibility of Holstein Friesian to heat stress than Brown Swiss. Lacetera, Bernabucci (16) reported different responses of peripheral blood mononuclear cells to long-term exposure to *in vitro* simulated heat stress between these two breeds. Their results indicated a more adverse response in Brown Swiss than Holsteins, simulating high body temperature in vitro conditions, probably due to different production levels and coat color (17).

This study aimed to deepen knowledge about different responses among Holstein Friesian and Brown Swiss cows to elucidate how productive, physiological, oxidative, and inflammatory patterns may change during and after a short duration (4 days) natural heat wave between animals of the two different breeds, but similar for production and physiological status, managed identically on the same commercial farm, to minimize or nullify any confounding factors capable of influencing their responses. We hypothesize that, despite being managed under the same conditions and having similar production levels, Holstein Friesian and Brown Swiss cows will exhibit distinct physiological, oxidative, and inflammatory responses to short-term heat stress. Specifically, we expect Holstein Friesian cows to show greater physiological alterations due to their known higher susceptibility to heat stress, while Brown Swiss cows may display a more stable response.

## 2 Materials and methods

All animal use and procedures for this study were approved by the Ethical Committee for Animal Welfare of Animals Employed in Scientific Research at the Department of Veterinary Medicine, University of Bari (approval no. 05/2022). The study was conducted in accordance with EU Directive 2010/63/EU on the protection of animals used for scientific purposes, Council Directive 98/58/EC on the protection of animals kept for farming purposes, and other relevant national and institutional guidelines.

### 2.1 Experimental design

**Figure 1:**
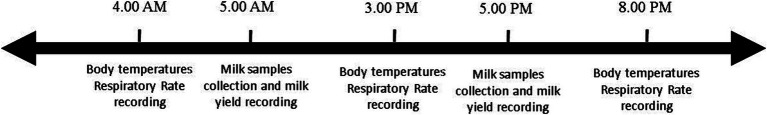
Temperature humidity index (THI) of Brown Swiss and Holstein cows exposed to a natural heat wave of 4 days with an absence of active cooling. Trend 2 days before starting the switch-off of the cooling system, up to the end of the trial (4 days after the switch-off) of THI in thermoneutrality (TN), corresponding to D1 at 4 AM (blood sampling) and heat stress (HS) corresponding to D4 at 3 PM (blood sampling), from 2 days before the start of the trial to D4.

The in-field trial was carried out from the 19th to the 22nd of July 2022 in a commercial dairy farm (Azienda Bruna Nuova di Maellaro, Noci, Italy; 40°43′53.4”N 17°06′50.1″E). Forty multiparous Brown Swiss (*n* = 20) and Holstein (*n* = 20) dairy cows were included. Groups were balanced for parity (Holstein = 2.80 ± 0.89, Brown Swiss = 2.60 ± 0.75, mean ± SD), days in milk (DIM; Holstein = 106.40 ± 9.12, Brown Swiss = 102.60 ± 7.30, mean ± SD) and body condition score (BCS; Holstein = 2.41 ± 0.12, Brown Swiss = 2.67 ± 0.15; mean ± SD). BCS was measured according to the scale grading of Ferguson et al. (1994). The Somatic Cell Score (SCS) for both groups was calculated using the formula SCS = log_2_(SCC/100) + 3 (Holstein = 3.91 ± 0.05, Brown Swiss = 3.82 ± 0.05; mean ± SD). All animals were fed the same TMR (Table 1). Every day refusal was measured for each group with the aim of mean dry matter intake calculation. The refusal was always less than 2% of the total administered. The study was conducted in a closed barn facility, where the animals were housed indoors and did not have access to an outside paddock. The barn was equipped with a roof that provided protection from solar radiation, covering both the resting area with cubicles and the feeding alley. The experimental treatment applied was a 4-day natural heat wave. The heat wave was not the first of the season to which the animals may have been subjected, but all animals had been exposed to the same conditions previously. Indeed, monitoring systems confirmed a THI below heat stress threshold levels in the 4 days preceding the experimental period (Figure 1). All the animals were kept in the same barn with access to shaded, straw-bedded free stalls. The barn had automated water soakers and fans (activation was set at THI of 72, calculated every 30 min). Soakers were located in the feeding area, while fans were in both feeding and resting areas. Barn environmental temperature and relative humidity (RH) were recorded every 5 min during the trial period with four data loggers (Hobo Pro series Temp probes, Onset Computer Corp., Pocasset, MA, United States). Two were placed in two different cubicles, and two in different positions in the feeding area (all of them were positioned at the height of the animal’s head). Dry bulb temperature and RH recorded by dataloggers helped calculate THI according to the following formula (18):


$$THI=\left(1.8\times AT+32\right)-\left(0.55–0.0055\times RH\right)\times \left[\left(1.8\times AT+32\right)-58\right]\text{,}$$

**Table 1:** TMR chemical composition.

Feed,^1^ kg/head	
Multigrass meadows hay	10.50
Mixed feed^2^	7.85
Corn meal	7.00
Wheat flour middling	0.30
Sugar cane molasses	0.30
Nutrients, % on DM basis	
DM	88.40
CP	15.69
aNDFom^3^	38.52
ADF	22.08
Ether extract	3.13
Ash	6.52
Starch	24.82

1 Wet basis.

2 Composition: extruded whole soybean, toasted and extruded soybean meal, barley flour, cotton seed, hulled sunflower flour, wheat flour, wheat bran, corn gluten feed, calcium carbonate, sodium chloride, dicalcium phosphate, calcium and magnesium carbonate, magnesium oxide, sodium bicarbonate, magnesium sulfate. Supplements per kg: vitamin A 54,000 UI; vitamin D3 6,000 UI; vitamin E 180 mg; choline chloride 270 mg; niacin 270 mg; betaine 60 mg; biotin 0.24 mg; calcium D pantothenate 6 mg; vitamin B1 6.0 mg; vitamin B2 6.0 mg; vitamin B12 0.06 mg; vitamin B6 3.0 mg; trace elements: Fe (iron carbonate) 34.44 mg; Fe (iron chelate) 6.13 mg; Mn 120.0 mg; I (potassium iodide) 1.26 mg; Cu (copper chelate of glycine-solid hydrate) 28.2 mg; Se (sodium selenite) 0.6 mg; Zn (zinc sulfate monohydrate) 178.8 mg; bentonite 6,000 mg.

3 Neutral detergent fiber analyses corrected for residual ash content.

where AT is the environmental temperature expressed in degrees Celsius, so that the term (1.8 × AT + 32) represents the conversion of temperature data in degrees Fahrenheit, and RH is the relative humidity as a fraction of unit. The mean THI recorded simultaneously by each of the four dataloggers was considered for further data analysis (Figure 1). The heat wave period was determined based on weather forecasts to ensure a THI exceeding 72. The start of the heat wave was considered as the moment when the cooling systems were turned off, leading to THI values surpassing 72.

All coolers and fans were switched off at the start of the trial and remained switched off for 4 consecutive days. At 04:00 AM, the coolest moment of the day before turning off the cooling systems was considered as a thermal neutral (TN) condition. The end of the 4 days, at 03:00 PM, the hottest moment of the day, was considered as a heat stress (HS) condition. All blood samples were collected at TN and HS times (Figure 1).

### 2.2 Physiological data

**Figure 2:**
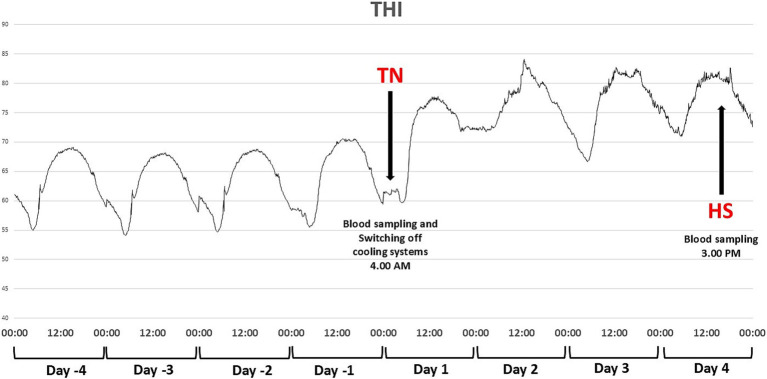
Experimental design and sampling scheme of Brown Swiss and Holstein cows exposed to a natural heat wave of 4 days with an absence of active cooling. Body temperatures and respiratory rates were collected daily at 4 AM, 3 PM, and 8 PM from D1 to D4; milk samples were collected daily at 5 AM and 5 PM from D7 to D4.

All physiological data were collected daily at 4:00 AM, 3:00 PM, and 8:00 PM (from day 1 to day 4; Figure 2). After capturing a cow in headlock gates, the respiration rate (RR) was visually measured by an expert assessor, who observed the movement of the animal’s rib cage for 30 s, then measures were doubled to be quantified as breaths per minute (bpm), rectal temperatures (RT) were measured by a digital rectal thermometer (Scala SC 1080, Stahnsdorf, Germany). Conjunctiva (CT), muzzle (MT), skin (ST); (measured on the tip of the ilium), and vulva skin (VT) temperature images were measured with a portable infrared thermography camera (ThermaCam i70 0, FLIR Systems AB, Danderyd, Sweden). To calibrate the camera results, environmental temperature and relative humidity were recorded with a digital thermo-hygrometer (Extech^®^ 44,550, Waltham, Massachusetts, United States). An image analysis software Therma Cam Researcher Pro 2.8 SR-2 (FLIR Systems AB, Taby, Sweden), was used to estimate temperatures, measuring the maximum temperature (°C) within an oval area traced around the anatomical site. Two images were taken for each measurement, and the mean values of both photos were used to estimate the temperature at that site. This maximum temperature was used for analyses.

### 2.3 Milk yield, sampling, and analysis

Milking was carried out in a milking parlor, and milk yield and milk sampling were carried out at each milking session from each cow (at 5:00 AM and 5:00 PM, respectively; Figure 2). From each cow, at each milking session, 100 mL of milk was sampled at the end of each milking session, which is representative of the entire milking yield. The samples where then refrigerated at 4°C, transported to the laboratory, mixed proportionally to the milk yields of the two daily milking sessions, and analyzed within 2 h, with near-infrared spectroscopy for fat, protein, casein, lactose, short-chain fatty acids (SFA), unsaturated fatty acids (UFA), monounsaturated fatty acids (MUFA), polyunsaturated fatty acids (PUFA) (19). Measures of milk coagulation properties (curd firmness, clotting velocity and renneting time) were obtained using the Formagraph instrument by Foss Electric A/S according to the procedure described in Dadousis et al. (20).

From these data, fat corrected milk (FCM) yield, standardized at 4% fat, was calculated according to the following formula (21):


4%FCM=0.4×milk+15×fat.

Moreover, the energy-corrected milk (ECM) yield was calculated according to the formula reported by Landi, Maggiolino (22):


$$ECM=\mathrm{milk}\times \left[0.25+\left(0.122\times \%\mathrm{fat}\right)+\left(0.077\times \%\mathrm{protein}\right)\right]$$

### 2.4 Blood sampling and analysis

Blood was drawn from the coccygeal vein into sterile Vacutainer tubes (Becton Dickinson and Co., Franklin Lakes, New Jersey, United States). At each thermal condition (TN and HS), two 9-mL lithium-heparin and two 9-mL clot activator tubes were collected from each cow. All the tubes were centrifuged at the farm, and serum and plasma were immediately frozen and stored at −20°C for further analysis.

Serum biochemical traits were measured using an automated biochemistry analyzer (CS-300B; Dirui, Changchun, China) (23). The following parameters were measured: alanine aminotransferase (ALT), aspartate aminotransferase (AST), alkaline phosphatase (ALP), glucose (Glu), blood urea nitrogen (BUN), creatinine (Crea), total serum protein (TP), albumin (Alb), cholesterol (Chol), triglycerides (Trig), uric acid, non-esterified fatty acids (NEFA), calcium (Ca), phosphorus (P), magnesium (Mg), chloride (Cl), (Gesan Production Kit, Campobello di Mazara, Trapani, Italy). In addition, globulins (Glob) were calculated using the TP and Alb parameters. Before each analytical session, the standards furnished in the assay kits were used to calibrate the multi-parameter analyzer (Seracal, Gesan Production Kit, Campobello di Mazara, Trapani, Italy). After setting the calibration curve, two multi-parameter control sera (Seracontrol N and Seracontrol P, Gesan Production Kit, Campobello di Mazara, Trapani, Italy) were tested to verify internal accuracy, considered satisfactory when the measured value deviated by no more than 3.00% from the manufacturer’s declared values (24). Sodium (Na) and potassium (K) were determined using the PennyVet chem 30 (EOS Italy, Milano, Italy) by method ISE indirect Na-K-Cl for Gen.2 (Roche/Hitachi cobas 8,000 ISE). Each sample was analyzed in triplicate, and the value used for the raw data set was the mean of the three recordings for each item analyzed.

#### 2.4.1 Plasma oxidative profile and total antioxidant capacity

Plasma (0.5 mL) was placed in a 50-mL test tube and homogenized with 15 mL deionized distilled water (DDW). Homogenate (1 mL) was transferred to a glass tube for the thiobarbituric acid reactive substances (TBARS) determination (25) and 0.05 mL of butylated hydroxytoluene (7.2% in ethanol) was added along with 1.950 mL of thiobarbituric acid (TBA)/trichloroacetic acid (TCA)/HCl (0.375% TBA, 15% TCA, and 0.25 N HCl). The sample solution was shaken and then incubated at 90°C for 15 min in a thermostatic bath. After this period, the samples were cooled to room temperature (15–30°C) and centrifuged at 2000 × g for 15 min. Supernatant absorbance at 531 nm was measured against a blank containing 2 mL of TBA/TCA/HCl solution in 1 mL of distilled water. The TBARS were calculated using a standard curve of 1,1,3,3-tetramethoxypropane. The concentration of lipid oxidation is expressed as milligrams of malondialdehyde (MDA) per mL of plasma.

For the determination of hydroperoxides, 0.5 mL of plasma was added to 4 mL of CH_3_OH and 2 mL of CHCl_3_, as described by (26). The samples were vortexed for 30 s and added to 2 mL of CHCl_3_ and 1.6 mL of 0.9% NaCl. The samples were shaken for 1 min and then centrifuged at 3500 × g for 10 min at 4°C. Two mL of lipid extract were sampled from the lower chloroform phase and then processed with 1 mL of CH_3_COOH/CHCl_3_ and 50 μL of KI (1.2 g/L mL distilled water). Samples were stored for 5 min in a dark room, added with 3 mL of 0.5% CH_3_COOCd, and then vortexed and centrifuged at 4500 × g for 10 min at 40°C. Absorbance at 353 nm was measured against a blank tube in which plasma was replaced by 2 mL of distilled water. The results are expressed in micromoles per mL.

Plasma (0.5 mL) was placed in 20 mL of 0.15 M KCl for 2 min. Two homogenate aliquots (50 μL each) were added with 1 mL 10% TCA and then centrifuged at 1200 × g for 3 min. at 4°C to measure protein carbonyls (27). The first aliquot was used as a standard and added with 1 mL of 2 M HCl solution. The second aliquot was added with 1 mL of 2 M HCl containing 10 mM 2,4-dinitrophenyl hydrazine (DNPH). The samples were incubated for 1 h at room temperature (15 to 30°C) and shaken every 20 min. Then, 1 mL of 10% TCA was added, samples were vortexed for 30 s, centrifuged three times at 1200 × g for 3 min at 4°C and the supernatant was removed without disrupting the pellet. The pellet was washed with 1 mL of ethanol: ethyl acetate (1:1), shaken and centrifuged three times at 1200 × g for 3 min at 4°C and the supernatant removed. The pellet was then dissolved in 1 mL 20 mM sodium phosphate 6 M guanidine hydrochloride buffer. The samples were then shaken and centrifuged at 1200 × g for 3 min at 4°C. Carbonyl concentration was calculated on the DNPH treated sample at 360 nm with a Beckman Coulter DU800 (Beckman Instruments Inc., Brea, CA, United States) and expressed as nanomoles of carbonyls per milligrams of protein. Proteins concentration was determined using the Biuret assay.

The ferric-reducing ability of the plasma (FRAP) assay was assessed to estimate the total antioxidant potential described by Benzie and Strain (28) with slight modifications. Three mL of freshly prepared FRAP reagent (1 mL of a 10 mM TPTZ solution in 40 mM HCl plus 1 mL of 20 mM FeCl3 in 10 mL H_2_O solution and 10 mL of 300 mM acetate buffer, pH 3.6) was incubated at 37°C for 40 min after mixing with 100 μL of plasma sample or supernatant. The absorbance of the reaction mixture was recorded at 593 nm, and the antioxidant power was expressed as μmol Trolox equivalents/mL. Radical scavenging activity of 2,2′-Azino-bis [3-ethylbenzothiazoline-6-sulphonic acid] (ABTS) was detected according to the procedure previously described by Re, Pellegrini (29) with some modifications. Briefly, ABTS radical cation was produced by mixing 7 mM ABTS stock solution with 2.45 mM potassium persulfate and keeping the mixture in the dark at 25°C for 12 to 16 h. The solution was then diluted in PBS to reach an absorbance value of 0.70 ± 0.02 at 734 nm. Then, 10 μL of plasma sample was added to 990 μL of diluted ABTS radical cation solution and incubated at 30°C for 5 min. The reagent blank was prepared by adding 10 μL of PBS instead of the sample. The scavenging of the ABTS radical cation was determined spectrophotometrically at 734 nm. Antioxidant activity was expressed as a percentage inhibition of ABTS radical cation and calculated by the following equation:


$$\begin{array}{l} \%\mathrm{inhibition}=100\times \left(\begin{array}{l} \mathrm{Absorbance}734\;\mathrm{Control}-\\ \mathrm{Absorbance}734\;\mathrm{Sample} \end{array}\right)/\\ \mathrm{Absorbance}734\;\mathrm{Control}\text{.} \end{array}$$

A clinical autoanalyzer (ILAB-650, Instrumentation Laboratory, Bedford, MA, United States) was used to determine the concentration of ceruloplasmin, reactive oxygen metabolites (ROMs) and paraoxonase (PON). The ROM were determined with a Diacron International srl kit (30). Retinol, tocopherol, and *β*-carotene concentrations were analyzed by reversed-phase HPLC (LC-4000, Jasco Europe SRL, Cremella, Italy) (31). Each sample analysis was performed in triplicate.

#### 2.4.2 Serum amyloid a, haptoglobin, heat shock protein 70, ceruloplasmin and paraxonase determination

The plasma samples were used for estimation of serum amyloid A (SAA, μg/mL), haptoglobulin (HP, mg/mL), heat shock protein 70 (HSP70, ng/mL; Cloud-Clone corporation, Katy, TX, United States) following the guidelines provided by the manufacturer and employing an automated microplate ELISA reader (Tecan Detection Infinite200 Pro). The plates were read at 450 nm. Ceruloplasmin was determined using the method proposed by Sunderman and Nomoto (32). Paraoxonase was determined by Ferré, Tous (33) and adapted to the ILAB 650, as described by Bionaz, Trevisi (34). All samples were tested in triplicate.

### 2.5 Statistical analysis

The freeware software Lenth, R. V. (2006–9) was used to determine the minimum sample size. (Retrieved 8/04/2022, from http://www.stat.uiowa.edu/~rlenth/Power.). The experimental design considered the 2 breeds and the 12 times, and for sample calculation, the value of *α* was set to 0.05 and *β* to 0.20, for a power of 0.90. The expected difference was set at 0.15. The rectal temperature and its standard deviation (0.14°C) were the outcome considered for sample size calculation (35). The sample size obtained was 18 cows, but we included 20 animals, considering the possibility of exclusion of some of them during the trial.

All data were analyzed using Shapiro–Wilk tests for normal distribution, and HSP70, HP, and SAA revealed deviation from normality. These data were normalized throughout the Log transformation, and then, as for all the other investigated parameters, the parametric statistic was applied. After statistical analysis, data were reconverted.

All parameters were analyzed by general linear mixed models using PROC MIXED with fixed effects of breed, time, and their interaction according to the following model:


$$Y_{\mathrm{ijkl}}=\mu +\alpha_{i}+B_{j}+T_{k}+{\left(B\times T\right)}_{jk}+\varepsilon_{ijk}$$

where Y_ijk_ represents investigated parameter as the dependent variable, μ is the overall mean; αi is the i^th^ dairy cow random effect within breed [i = 1,…20; the variance–covariance structure chosen and applied was Compound Symmetry (CS)], B_j_ is the effect of the j^th^ breed (j = 1, 2), T_k_ is the effect of the T^th^ time (k = 1, …5 for milk parameters; k = 1,…12 for rectal temperature, RR, and thermo-camera data; k = 1, 2 for biochemical, oxidative, and inflammatory parameters), (B × T)_jk_ is the binary interaction between the j^th^ breed and the T^th^ time (jk = 1,…10 for milk parameters; jk = 1,…24 for rectal temperature, RR, and thermo-camera data; jk = 1,…4 for biochemical, oxidative, and inflammatory parameters) and ε_ijk_ is the error term.

Thermal conditions were subdivided and assessed as TN, corresponding to D1 at 4 AM, and HS, corresponding to D4 at 3 PM, and these two timepoints were considered in the model for biochemical, oxidative, and inflammatory parameters.

A pairwise comparison between breeds within same time was performed using the Bonferroni test. A Tukey test was applied to evaluate the differences among means within the same breed when the effect of time or the binary interaction of treatment × time was significant. Significance was set at *p* < 0.05; the results are expressed as least square means and standard error of the means. All the analyses were performed using SAS software package (36).

## 3 Results

THI data from D-4 to D4 of the trial are presented in Figure 2, considering D1 the day the onset of heat wave at 8:00 AM. Temperature humidity index ranged from about 55 in the early morning to about 68 at 12:00, 2 days before the switch off. After turning off the cooling systems, THI peaked at 85 at 12:00 of D2, with the lowest value of about 67 at midnight of D3. During the 4 days, maximum THI values were 77.8, 84.1, 82.7, and 82.6, respectively; minimum daily THIs recorded were 59.6, 71.8, 66.7, and 71.0, respectively.

**Figure 3:**
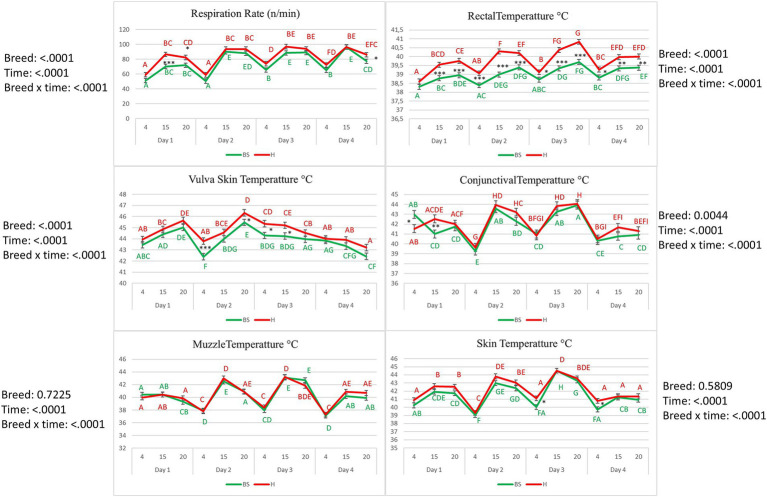
Rectal temperature (°C), respiration rate (breaths/min), conjunctival temperature (°C), muzzle temperature (°C), vulva skin temperature (°C), and skin temperature (°C) of the Brown Swiss (BS) cow and Holstein (H) experiencing a 4-day heat wave. Different letters of the same color correspond to a statistically significant difference within the same breed: A, B, C, D, E, F, G, H, I = *p* < 0.01. Asterisks correspond to a statistically significant difference between the breeds at the same Condition: *p < 0.05; **p < 0.01; ***p < 0.01.

Figure 3 shows results on the variation of RR and body temperatures in Brown Swiss and Holstein cows. The RR decreased to the lowest measured values at 4:00 AM every day of the trial in both breeds (*p* < 0.01). Basal levels at 4:00 AM were higher on D3 than on previous days in both breeds (*p* < 0.01). RT increased each day of the trial from 4:00 AM to 3:00 PM in both breeds. Holstein cows had higher RT values during the experimental period than Brown Swiss cows (*p* < 0.01). In both breeds, a rising trend was recorded on the first day for VT, with a decrease on D2 at 4:00 AM, followed by an increase until 8:00 PM on the same day. From 4:00 AM, at D3, the VT was declining (*p* < 0.01). Differences between breeds were found at 4:00 AM on D2 with higher values in Holsteins (*p* < 0.001) and from 8:00 PM of D2 to 3:00 PM of D3, also in this case with higher temperatures in Holsteins (*p* < 0.05). Conjunctival temperature in Brown Swiss was higher (*p* < 0.05) at 4:00 AM of D1 and lower (*p* < 0.01) than in Holsteins at 3:00 PM of the same day; from 8:00 PM of D1 to 8:00 PM of D4 conjunctival temperature showed no differences between the two breeds. Muzzle temperature increased at 3:00 PM on D2 and D3, declined at 8:00 PM at 4:00 AM in both the breeds. On D4, an increase in muzzle temperature was recorded at 3:00 PM (*p* < 0.01). Skin temperature in both breeds increased slightly on D1 from 4:00 AM to 8:00 PM, then decreased at 4:00 AM on D2, followed by an increase at 3:00 PM and decreased at 4:00 AM and 8:00 PM both on D2 and D3 (*p* < 0.01). Neither muzzle nor skin temperature showed differences between the breeds (*p* > 0.05).

Table 2 presents the effect of time and breed on some production traits. Brown Swiss had no significant milk yield (MY) modifications from D1 to D4, whereas in Holstein cows, MY decreased (*p* < 0.05) from D1 to D4. Therefore, FCM yield and ECM yield decreased (*p* < 0.05) on D4 compared to D1 and D2 in Holstein cows, while no differences were recorded in Brown Swiss. On the other hand, no changes across the days were found in both protein and casein concentration; Brown Swiss had higher values than Holsteins on D1, D2, and D3 (*p* < 0.05), as well as in D4 (*p* < 0.01). Fat, lactose, SFA, UFA, MUFA, and PUFA were not affected by breed or time. Higher curd firmness and lower clotting velocity values were recorded in Brown Swiss (*p* < 0.01) throughout the trial, with no changes across days. Additionally, Brown Swiss showed lower renneting time values (*p* < 0.01) on D1, D2, and D4. These parameters (curd firmness, clotting velocity, and renneting time) also differed between breeds in the pre-experimental samples (*p* < 0.01), and these breed-related differences were not affected during the heat wave.

**Table 2:** Milk production and quality traits in Brown Swiss and Holstein cows experiencing a 4-day heat wave.

Parameter	Breed	Mean value of 7 days before	Days				Analysis of variance			
			1	2	3	4	SEM^1^	B^2^	D^3^	B × D
Milk Yield (kg)	BS^13^	34.78	35.12	33.47	32.13	32.83	1.39	0.6455	0.0236	0.6061
	H^14^	36.23^a^	36.38^a^	35.5^ab^	32.31^b^	31.21^b^				
FCM^4^ yield (kg)	BS	42.35	42.51	38.9	39.14	38.67	1.93	0.9680	0.0307	0.4113
	H	42.45^a^	42.8^a^	42.41^a^	37.91	35.89^b^				
ECM^5^ yield (kg)	BS	38.99	38.92	35.58	35.81	35.37	1.77	0.8957	0.0310	0.4110
	H	38.79^a^	39.03^a^	38.71^a^	34.57	32.73^b^				
Fat (%)	BS	4.79	4.89	4.77	4.9	4.79	0.12	0.1275	0.7207	0.4841
	H	4.72	4.63	4.81	4.61	4.53				
Protein (%)	BS	3.41^x^	3.41^x^	3.42^x^	3.41^x^	3.41^X^	0.07	<0.0001	0.8092	0.9115
	H	3.17^y^	3.14^y^	3.19^y^	3.19^y^	3.11^Y^				
Casein (%)	BS	2.70^x^	2.71^x^	2.69^x^	2.68^x^	2.68^X^	0.06	<0.0001	0.9434	0.9555
	H	2.46^y^	2.48^y^	2.50^y^	2.49^y^	2.41^Y^				
Lactose (%)	BS	4.88	4.84	4.89	4.89	4.9	0.02	0.9810	0.3801	0.4019
	H	4.86	4.87	4.86	4.93	4.86				
SFA^6^ (%)	BS	3.20	3.22	3.14	3.22	3.13	0.08	0.2205	0.7118	0.4672
	H	3.12	3.04	3.15	2.98	2.98				
UFA^7^ (%)	BS	1.44	1.45	1.39	1.43	1.42	0.04	0.2414	0.7909	0.5655
	H	1.38	1.37	1.4	1.36	1.32				
MUFA^8^ (%)	BS	1.30	1.32	1.27	1.3	1.29	0.04	0.2353	0.8289	0.6431
	H	1.28	1.27	1.3	1.26	1.22				
PUFA^9^ (%)	BS	0.12	0.13	0.12	0.12	0.12	0.004	0.3108	0.6740	0.7136
	H	0.13	0.12	0.12	0.12	0.11				
A30^10^ (mm)	BS	24.05^X^	24.03^X^	22.34^X^	21.24^X^	22.56^X^	1.33	<0.0001	0.9813	0.2791
	H	14.02^Y^	13.37^Y^	14.48^Y^	15.77^Y^	13.74^Y^				
K20^11^ (min)	BS	8.01^X^	7.73^X^	8.15^X^	8.13^X^	7.86^X^	0.25	<0.0001	0.8807	0.2808
	H	9.68^Y^	9.76^Y^	9.56^Y^	9.21^Y^	9.51^Y^				
R^12^ (min)	BS	15.22^X^	14.22^X^	14.97^X^	15.35	14.18^X^	0.66	<0.0001	0.7477	0.1763
	H	17.41^Y^	18.06^Y^	18.1^Y^	16.6	17.93^Y^				

1 Standard Error of the Mean.

2 Breed.

3 Day.

4 Fat corrected milk.

5 Energy corrected milk.

6 Short-chain fatty acids.

7 Unsaturated fatty acids.

8 Monounsaturated fatty acids.

9 Polyunsaturated fatty acids.

10 Curd firmness.

11 Clotting velocity.

12 Renneting time.

13 Brown Swiss.

14 Holstein.

Different letters on the same line correspond to a statistically significant difference among the days within the same breed: a, b = *p* < 0.05. Different letters on the same column for each parameter correspond to a statistically significant between breeds within the same day: X, Y = *p* < 0.01; x, y = *p* < 0.05.

The results of serum biochemical analysis are reported in Table 3. Albumin showed higher (*p* < 0.05) values in Brown Swiss than in Holsteins under TN conditions, although no differences were detected among breeds in TP and Glob. A marked increase of BUN values under stress conditions was detected in both the breeds (*p* < 0.01). Uric acid, in contrast, showed lower (*p* < 0.01) values in the HS condition for both the breeds. Magnesium had higher values in HS conditions both in Brown Swiss (*p* < 0.05) and Holstein cows (*p* < 0.01). Higher ALT values (*p* < 0.05) were detected in Brown Swiss than in Holstein during HS. No significant differences were found for Crea, K, Gluc, AST, or ALP, considering both breed and environmental factors.

**Table 3:** Biochemical profile patterns of Brown Swiss and Holstein Friesian cows in thermoneutral conditions and heat stress after a 4-day duration heat wave.

Parameter	Breed	Condition		Analysis of variance			
		TN^1^	HS^2^	SEM^3^	B^4^	T^5^	B × T
Total Protein (g/L)	BS^11^	78.4	81.5	1.21	0.1918	0.2421	0.7040
	H^12^	80.6	82.7				
Albumin (g/L)	BS	36.2^x^	35.1	4.74	0.0252	0.5720	0.3982
	H	34.3^y^	33.6				
Globulins (g/L)	BS	43.6	45.7	1.14	0.2323	0.1616	0.9565
	H	46.8	48.8				
Creatinine (mmol/L)	BS	67.77	72.22	4.32	0.8752	0.2138	0.8642
	H	62.01	69.44				
BUN^6^ (mmol/L)	BS	2.81^A^	3.42^B^	0.02	0.2149	<0.0001	0.7043
	H	2.39^A^	3.11^B^				
Uric acid (g/L)	BS	2.44^A^	1.88^B^	0.07	0.3242	<0.0001	0.1045
	H	2.22^A^	1.84^B^				
Chloride (mmol/L)	BS	105.95	106.57	1.45	0.4868	0.3142	0.5412
	H	103.48	17.07				
Sodium (mmol/L)	BS	148.35	146.46	1.85	0.4367	0.3858	0.0562
	H	144.16	146.13				
Calcium (mmol/L)	BS	2.52	2.35	0.18	0.2934	0.4322	0.4050
	H	2.33	2.44				
Phosphorous (mmol/L)	BS	2.33	2.67	0.33	0.1522	0.4204	0.4727
	H	2.39	2.94				
Magnesium (mmol/L)	BS	1.15^a^	1.62^b^	0.02	0.8660	0.0005	0.3418
	H	1.30^A^	1.40^B^				
Potassium (mmol/L)	BS	4.24	4.20	0.46	0.0970	0.7661	0.5670
	H	4.13	4.14				
Cholesterol (mmol/L)	BS	9.54	9.33	1.3	0.2213	0.7836	0.9369
	H	8.12	8.23				
Triglycerides (mmol7L)	BS	0.25	0.24	0.77	0.8146	0.3358	0.5582
	H	0.29	0.23				
NEFA^7^ (mmol/L)	BS	0.15	0.13	0.01	0.6628	0.4248	0.3112
	H	0.14	0.13				
Glucose (mmol/L)	BS	3.36	3.30	1.85	0.5853	0.4763	0.1759
	H	3.28	3.49				
ALT^8^ (IU/L)	BS	22.15	24.91^x^	1.3	0.2052	0.6671	0.0923
	H	22.70	21.05^y^				
AST^9^ (IU/L)	BS	96.48	96.96	6.8	0.6299	0.7098	0.6581
	H	102.76	97.23				
ALP^10^ (IU/L)	BS	98.10	97.75	6.75	0.0304	0.6960	0.7330
	H	116.03	110.88				

1 Thermoneutrality, corresponding to D1 at 4 AM.

2 Heat stress, corresponding to D4 at 3 PM.

3 Standard Error of the Mean.

4 Breed.

5 Thermo condition.

6 Blood Urea Nitrogen.

7 Not Esterified Fatty Acids.

8 Alanine transaminase.

9 Aspartate aminotransferase.

10 Alkaline phosphatase.

11 Brown Swiss.

12 Holstein.

Different letters on the same line correspond to a statistically significant difference between conditions within the same breed: A, B = *p* < 0.01; a, b = *p* < 0.05. Different letters on the same column for each parameter correspond to a statistically significant between breeds within the same day: X, Y = *p* < 0.01; x, y = *p* < 0.05.

Some plasma biomarkers of inflammation and oxidative profile parameters are reported in Table 4. Brown Swiss cows had higher (*p* < 0.01) FRAP values than the Holstein breed in both conditions. Brown Swiss showed higher (*p* < 0.01) ABTS values during TN than under HS, and Holstein cows in TN conditions. Lower (*p* < 0.05) TBARs values were observed in Holstein cows under TN conditions compared with HS, and Brown Swiss in TN. Hydroperoxides increased (*p* < 0.01) in both breeds during HS.

**Table 4:** Oxidative and inflammatory patterns of Brown Swiss and Holstein Friesian cows in thermoneutral conditions and heat stress after a 4-day duration heat wave.

Parameter	Breed	Condition		Analysis of variance			
		TN^1^	HS^2^	SEM^3^	B^4^	T^5^	B × T
FRAP (μmol TE/ml)^6^	BS^14^	57.00^X^	55.00^X^	2.3	0.0007	0.2034	0.0077
	H^15^	30.52^Y^	40.38^Y^				
ABTS (%/l)^8^	BS	63.00^AX^	49.00^B^	2.4	0.0047	0.0034	0.1770
	H	47.00^Y^	48.00				
TBARs (mmol/ml)^7^	BS	0.74^x^	0.75	0.03	0.0470	0.0389	0.1412
	H	0.64^ay^	0.74^b^				
Hydroperoxides (mmol/ml)	BS	5.30^A^	6.16^B^	0.08	0.2442	<0.0001	0.1845
	H	5.01^A^	6.10^B^				
Protein Carbonyls (mmol/ml)	BS	100.11	98.31	1.48	0.7507	0.3029	0.2088
	H	98.66	99.45				
SAA (μg/mL)^9^	BS	2.27	1.78	0.19	0.4521	0.5589	0.3557
	H	2.78	1.71				
HP (mg/mL)^10^	BS	0.42^a^	0.55^bX^	0.03	0.6965	0.4328	0.0959
	H	0.41	0.34^Y^				
HSP70 (ng/mL)^11^	BS	147.10^a^	185.59^bx^	13.32	0.0007	0.0128	0.4547
	H	171.85^A^	230.96^By^				
Ceruloplasmin (μmol/L)	BS	2.67^X^	2.81^X^	0.12	<0.0001	0.6993	0.4927
	H	2.12^Y^	2.08^Y^				
ROM (mg of H_2_O_2_/100 mL)^12^	BS	12.75^x^	13.03^x^	0.66	0.0015	0.8254	0.8356
	H	10.69^y^	10.70^y^				
PON (U/mL)^13^	BS	70.85	73.67	3.49	0.8538	0.5116	0.8848
	H	70.71	72.51				
Retinol (μg/100 mL)	BS	41.46^x^	40.70	1.7	0.078	0.8822	0.7647
	H	36.34^y^	36.59				
Tocopherol (μg/mL)	BS	4.51^x^	4.92^X^	0.42	0.0006	0.4881	0.7962
	H	3.07^y^	3.26^Y^				
β-carotene (mg/100 mL)	BS	0.15	0.17^x^	0.01	0.0072	0.4045	0.7592
	H	0.11	0.12^y^				

1 Thermoneutrality, corresponding to D1 at 4 AM.

2 Heat stress, corresponding to D4 at 3 PM.

3 Standard Error of the Mean.

4 Breed.

5 Thermo condition.

6 Ferric-reducing antioxidant power assay.

7 Thiobarbituric acid reactive substances assay.

8 2,2′-azino-bis (3-ethylbenzothiazoline-6-sulfonic acid) assay.

9 Serum amyloid A.

10 Haptoglobin.

11 Heat Shock Protein 70 kilodaltons.

12 Reactive oxygen metabolites.

13 Paraoxonases.

14 Brown Swiss.

15 Holstein.

Different letters on the same line correspond to a statistically significant difference between conditions within the same breed: A, B = *p* < 0.01; a, b = *p* < 0.05. Different letters on the same column for each parameter correspond to a statistically significant between breeds within the same day: X, Y = *p* < 0.01; x, y = *p* < 0.05.

Higher plasma protein carbonyl concentrations were detected in the TN condition than in the HS condition in Brown Swiss (*p* < 0.01), and then Holstein cows in TN conditions (*p* < 0.05). In contrast, HS Brown Swiss cows had higher HP values (*p* < 0.05) compared with TN condition and to HS Holstein cows (*p* < 0.01). An increase of HSP70 was detected in both Brown Swiss (*p* < 0.05) and Holstein (*p* < 0.01) cows during HS, compared to the values of each breed under TN conditions. Moreover, with HS, the plasma concentration of HSP70 was higher in Holstein than in Brown Swiss (*p* < 0.05). Ceruloplasmin (*p* < 0.01) and ROM (*p* < 0.05) displayed higher values in Brown Swiss than in Holstein both in TN and HS conditions. Similarly, tocopherol was higher for Brown Swiss than Holstein cows both in TN (*p* < 0.05) and HS (*p* < 0.01) conditions. Brown Swiss showed higher values for retinol and *β*-carotene vs. Holsteins (*p* < 0.05) in TN and HS, respectively. No significant differences were observed in PON values.

## 4 Discussion

Hot environmental conditions compromise the well-being of animals, leading to various modifications that influence the physiological and production traits of dairy cows. The THI is commonly used to estimate the degree of HS in dairy cattle (37). However, THI does not directly measure HS. This index can predict stress in dairy cows according to some environmental parameters (temperature and relative humidity). In the present study, THI was higher than 72 for 96 h and over 74 for 78 h. Indeed, THI exceeded the upper critical THI threshold of 72 in Holstein (13) and 74 in Brown Swiss (2). Thus, both breeds in our study experienced a natural heat wave, typical of the Mediterranean region, during the summer. Looking at the weather data and considering the time of year when this experiment was carried out, this wasn’t the first heatwave that the animals involved were exposed to. Therefore, some variations of the investigated values may already be indicative of a metabolic change due to heat stress (i.e., urea, Alb, and some inflammatory parameters). Additionally, both breeds were kept in the same environment and fed in the same way, and there were no differences in BCS, ECM yield, and DIM. Considering these aspects, the present work, based on this experimental design, can highlight the breed effect better and more directly than other studies in which the animals involved differed in metabolic status or production level. Moreover, the trial was planned to assess both breeds’ short-term adaptative responses and not the responses to chronic HS or its medium-long-term effects. Rectal temperature, considered the gold standard for determining HS conditions, increased with the rising THI in the absence of heat abatement, displaying lower values at every 4:00 AM timepoint daily. Rectal temperature was higher in both breeds at 3:00 PM and 8:00 PM compared with 4:00 AM, with higher values in Holsteins. At 4:00 AM, both the breeds showed similar values. This means that a Holstein’s recovery capacity in lowering RT during the night was faster relative to Brown Swiss. These results are partially contradictory to those reported by others (11). Although throughout the experimental trial, the Holstein showed a higher RT compared to the Brown Swiss, it is not equally valid that the Holstein had less capacity for heat dissipation, as reported by Mylostyvyi et al. (38), given the significant nocturnal recovery observed.

However, it is important to compare the measured RT values with established thresholds to confirm whether the cows experienced heat stress. The normal RT range for dairy cows is between 38.0 and 39.3°C (39), with values above 39.5°C typically indicating heat stress. In our study, RT values during the hottest hours of the day approached or exceeded this threshold, supporting the conclusion that the cows were under heat stress. The observed increase in RT reflects a physiological response to thermal strain, consistent with previous findings in similar climatic conditions (40). Furthermore, the gradual rise in RT over the four-day heat wave suggests an accumulating heat load in the animals, further emphasizing RT’s role as a key indicator of heat stress intensity in dairy cows. In the attempt to dissipate heat, animals increased RR to maintain thermal equilibrium (41) indeed, this parameter has been identified as useful in predicting and assessing heat stress severity (42). Some authors suggested an RR threshold (between 65 and 68) for detecting HS in lactating cows (43), confirming that cows involved in this trial underwent heat stress suddenly on the first day. However, the literature reported contrasting results regarding RR in the two different breeds. Moore et al. (44) observed higher values of RR in HS Brown Swiss compared with Holstein cows, while conversely Correa-Calderon et al. (11) reported that Holsteins are characterized by higher RR relative to Brown Swiss under heat-stress conditions. However, it should be emphasized that, despite a difference in RT between the two breeds, this did not manifest an equally marked difference in RR. This let hypothesize that there are no differences between Brown Swiss and Holstein cows in terms of heat dissipation capacity through panting.

Furthermore, both RT and RR showed an increasing trend over the 4 days of the heat wave, indicating an effect of heat load on cows. Skin temperature and conjunctival temperature showed a similar trend to RT, with no differences between breeds. Skin temperature is a highly sensitive trait in response to HS, and it represents an important pathway for heat exchange, and skin temperature is the result of the regulation of this exchange between the skin and body core by the blood flow (45). This means that, despite differences in RT, this lack of breed effect on skin temperature supports the hypothesis that there were no differences in heat dissipation between the breeds during a short-term HS exposure. Conjunctiva temperature reflects core body temperature in cows well (46), and is correlated to RT (42). However, it should be noted that the barn was not completely enclosed, allowing for the possibility of direct solar radiation exposure, particularly when the sun’s rays were not perpendicular to the ground. While the cubicles and feeding alley were covered to provide shade, cows could still position themselves in areas where they received direct radiation. This factor could have influenced the absolute values of skin temperature measurements, despite careful calibration of the IRT camera. Therefore, while the trends observed in skin temperature remain valid, the absolute values should be interpreted with caution, considering the potential effect of solar radiation exposure. Muzzle and VT showed a different trend compared with RT. Muzzle temperature decreased the first day and reached the highest values at 3:00 PM on the other days, confirming the low correlation reported by George, Godfrey (47) between muzzle and RT. In contrast, VT increased until the second day and then decreased slowly.

The alteration of RR and RT parameters can influence the productive capacity of cows (40, 48). Many authors have concluded that HS affects high-producing cows more because the thermal neutrality zone shifts to lower temperatures as milk production, feed intake, and metabolic heat production increase (49, 50). While Italian Brown Swiss cattle are overall less productive than Holstein Friesians [i.e. mean lactation production of 7,500—(51)^1^ vs. 10,386 kg—(52)^2^], meaning their heat sensitivity is less affected by milk yield, it is important to highlight that animals of both breeds chosen for our study had the same production levels. Indeed, the two groups had the same energy expenditure for production, with no differences in ECM prior to the heat wave. This further supports the idea that the lack of significant differences between the breeds in physiological pathways attempting to respond to an increase in heat load could be consistent with no difference in the energy expenditure faced for milk production. Furthermore, Brown Swiss cows seldom decreased MY (2), unlike Holstein cows (13) under HS conditions. Even in such a scenario of a short heat wave, Brown Swiss cows tended to show about half the reduction (−9% for FCM, −7% for ECM and −5.6% as MY) in comparison with Holsteins (−15% for FCM, 15% for ECM and 13% for MY). No variations were found in both breeds for fat, SFA, UFA, MUFA, and PUFA percentages, consistent with results noted by Bernabucci, Biffani (13) in Holsteins and Maggiolino, Dahl (2) in Brown Swiss. The higher protein and casein percentage in Brown Swiss over Holstein cattle can be explained by physiological differences between breeds (53), and the lack of variations during the days implies that there was no persistent influence of only 4 days of HS on these two parameters. Lactose percentage showed no changes, consistent with (54). Curd-firmness was higher in Brown Swiss cows, highlighting the better milk processing characteristics of this breed compared to Holsteins, which enables farmers to enhance the entire value chain for cheese production (55). The Holstein cows showed a decrease in milk yield, confirming what has been reported in the literature (13, 48), although they never exhibited MY values lower than the Brown Swiss. The reduction in MY represents a physiological outcome aimed at counteracting HS, which partially contrasts with the RT pattern in Holstein cows, where the relative variation in temperature was greater compared to Brown Swiss, reflecting a higher capacity for temperature recovery. Milk yield, also normalized to ECM, declined over the 4 days of observation. This decrease was recorded in both the breeds, with higher losses in Holstein cows. These results are consistent with findings by West (39), who reported that dairy cows exposed to heat stress often exhibit reduced MY due to a combination of decreased feed intake and altered physiological responses. The larger decline in Holsteins compared to Brown Swiss could be attributed to breed-specific differences in thermoregulation and metabolic responses to heat stress, as noted by Bouraoui, Lahmar (56).

The blood protein profile was not affected by short-term HS in either breed, although the albumin levels of Holsteins is at the lower border line level of clinically healthy cows for their lactation stage (57, 58). This could suggest that Holstein cows were already under a mild type of stress. Some authors have reported that a reduction in blood levels of albumin may be linked to immunodeficiency, inflammation, or hepatic diseases (59); and an increase can occur under dehydration conditions (60). The absence of variation in the protein profile confirmed that it is not an appropriate short-term HS index if proper water intake is provided to ensure an adequate state of hydration. Even creatinine, reported in the literature to increase in the case of HS (61), did not vary in significant manner between breeds, but increased in both breeds about 7% in 4 days, probably due to short-term exposure to HS. Interestingly, there were not significant changes in NEFA levels over the four-day period in both breeds. NEFA levels are feasible patterns for detecting fat depot mobilization for supporting the energy demand, a metabolic pathway usually activated during a negative energy balance experienced by cows for avoiding the drop of productivity. We did not record a significant modification in blood NEFA concentration in either breed, despite the likely energy shortfall caused by heat stress. This outcome aligns with the findings of Bernabucci et al. (41) and Rhoads et al. (62), who also observed that NEFA levels did not increase under heat stress conditions. Our results suggest different hypotheses, considering that the two experimental groups started the trial with the same energy expenditure for milk production, as the ECM yield did not differ. Probably, the two breeds have a different energetic demand for the physiological responses to cope with heat stress, or a different energy partitioning, considering the differences in body temperatures we recorded. On one hand, there could be differences, not highlighted in this study, related to decrease in dry matter intake that occurs during heat stress (63). Although the present study provides valuable insights, it was not designed to answer to these questions, therefore, further research is needed to better focus on increase knowledge on the different responses between these breeds in energy balance and partitioning during heat stress conditions.

The electrolyte profile does not show differences in heat stress due to the short-term HS, although some differences between Brown Swiss and Holsteins were observed. However, their values always remained within the physiological ranges. Electrolyte and acid–base imbalances are generally associated with increased sweating and respiratory rate (64). During HS, K^+^ loss through sweat is substantial, and its secretion intensifies with prolonged exposure and rising temperatures (50). Na^+^ is also excreted through the skin, albeit at a lower rate than potassium, and its elimination similarly increases over time (64). In our case, the relatively short duration of heat stress may have contributed to the stability of these parameters.

Oxidative stress is an imbalance between antioxidants and oxidative molecules, such as reactive oxygen species and lipid peroxides (65). The production of metabolic heat and oxidative responses intensifies as thermal stress increases (66, 67), leading to metabolic disorders and greater susceptibility to infectious diseases (65). Production of oxygen-derived free radicals increases with HS, causing a reduction in antioxidant activity (68), resulting in some of the negative effects associated with HS (69, 70). Our results showed a decrease in ABTS only in Brown Swiss and an increase in hydroperoxides after exposure to HS, with slight differences between breeds and no differences in other oxidative parameters, or reactive oxygen metabolites (ROMs). Previous studies have shown reduced levels of total antioxidant capacity and superoxide dismutase in heat-stressed cows, as well as increased lipid peroxidation leading to oxidative stress (66, 71, 72), but evidently, the short-term exposure to HS was not sufficient to induce significant changes in the oxidative balance of either breed.

Plasma haptoglobin increases in dairy cows during high environmental temperatures (73), as confirmed in our results in Brown Swiss cows. Haptoglobin is a major acute-phase proteins in cow to assess the health and inflammatory response of animals (74); it is released from hepatocytes in response to tissue injury or infection (75) and acts as a potent antioxidant as well as a potent suppressor of lymphocyte function (76). Moreover, HP is released from many tissue mucosae, which are much more exposed to HS than liver (77). It has been demonstrated that HP values increase due to thermal stress, showing the relationship between the changes and the different climatic conditions (73). However, these studies investigated the seasonal effect, with cows exposed to HS for a longer time in a different experimental condition than the one described in this study. Moreover, HP levels were already high at the beginning of the heat wave (i.e., > 0.2 g/L) (57) suggesting a previous inflammatory challenge. Acute inflammatory processes are usually reversible, whereas chronic inflammation has a poor prognosis. It is clinically important to distinguish between these two states, typically based on the duration of the disease and the results of clinical chemistry and hematological investigations (78). However, the disease duration is often unknown, and there are limited clinical variables to differentiate between acute and chronic inflammation. In this context, HP, as a major acute-phase protein, could reflect such inflammatory challenges, even before the onset of heat stress. Serum Amyloid A is the major bovine acute phase protein. It is an apolipoprotein mainly expressed in the liver, and is the major acute phase protein overproduced during the inflammatory acute phase response (74). It is over-expressed in several disorders; thus, changes in its concentration are considered blood-based protein inflammation biomarkers in adult cows (79). Remarkably, the serum concentration of SAA during HS does not change outside of the physiological range (80, 81). This is consistent with studies showing that while SAA levels often rise in acute inflammatory conditions, in cases of heat stress, it may not necessarily reflect the same degree of inflammation as other markers like HP. This suggests a possible difference in the cytokine response, such as a lower production of pro-inflammatory cytokines during heat stress compared to other acute inflammatory states (78). Heat shock protein 70 (HSP70) is the most sensitive among all the heat shock proteins, and it is considered an important regulator of thermal adaptation during HS in livestock (82). Brown Swiss and Holstein cows increased HSP70 values earlier than other parameters in the present study. HSP70 promotes the proper folding of newly synthesized proteins but also aids in returning denatured proteins to their native state, facilitating livestock acclimatization to thermal stress (83). It stimulates cellular survival, playing a critical role in acquiring thermotolerance in livestock (84) and is essential for coping with thermal stress at the cellular level in sheep (85), goats (86), cattle and buffalo (83). In our trial, other stress indicators, such as ceruloplasmin, ROM, PON, and retinol (considered as the index of retinol binding protein), showed no significant impact due to HS, even if certain ones showed significant differences between breeds before of the heat wave (i.e., ceruloplasmin, ROM), further evidence that Brown Swiss and Holstein demonstrate differences in the use of some nutrients and in the immune response according to breed (i.e., higher levels of ceruloplasmin and ROM) (87). Similar results were reported for tocopherol and *β*-carotene, indicating that short term HS did not affect their plasma levels in dairy cows. Again, the brief exposure to heat stress conditions, both in terms of intensity and duration, may account for the lack of effect in our study. This suggests that such short-term exposure might not have been sufficient to induce variations in these parameters within the same breed. Although our study provides valuable insights, there are several limitations that should be considered when interpreting the results. First, the relatively short duration of heat stress exposure may not have been sufficient to induce significant physiological changes across all parameters, which could have limited the extent of the observed effects. Additionally, the intensity of the heat stress conditions, while designed to mimic natural environmental stress, may not have been as severe as those experienced in prolonged heat waves, which could affect the generalizability of our findings. Furthermore, the absence of changes between Brown Swiss and Holsteins implies that the pathway control system in these two breeds is similar.

## 5 Conclusion

This study compared the responses of Holstein and Brown Swiss cows to a 4-day heat wave under identical conditions. Holstein cows showed higher RT, especially in the evening, with a greater overnight recovery, while other physiological parameters (RR, ST, VT, CT, MT) did not show clear breed differences. HSP70 was the only parameter that increased as expected in both breeds. The elevated HP and albumin levels suggest prior heat exposure may have influenced the response. These findings highlight the need for further studies with prolonged heat stress to better understand breed-specific adaptation mechanisms.

## Data Availability

The raw data supporting the conclusions of this article will be made available by the authors, without undue reservation.

## Footnotes

^1^ https://www.anarb.it/en/home/

^2^ https://www.anafi.it/en
